# Subsoil tillage improved the maize stalk lodging resistance under high planting density

**DOI:** 10.3389/fpls.2024.1396182

**Published:** 2024-07-17

**Authors:** Xueying Feng, Daling Ma, Tianen Lei, Shuping Hu, Xiaofang Yu, Julin Gao

**Affiliations:** Agricultural College, Inner Mongolia Agricultural University, Hohhot, China

**Keywords:** maize, subsoil tillage, stalk morphology, stalk strength, lodging rate

## Abstract

Lodging reduces maize yield and quality. The improvement in maize lodging resistance has proven to be instrumental in maximizing the yield potential of maize varieties under high-density planting. Tillage practices accommodate larger groups by enhancing soil conditions. This study aimed to elucidate the impact of subsoil tillage in reducing the maize stalk lodging rate. The maize cultivars Xianyu 335 (XY335) and Zhongdan2 (ZD2) were selected for field experiments including two tillage methods, shallow rotary (RT) and subsoil (SS), and two densities, 75,000 plants ha^−1^ (D1) and 105,000 plants ha^−1^ (D2), were set up to investigate and analyze the changes of maize lodging rate and the related indexes of lodging resistance under SS and RT conditions. The findings revealed that under high density, as compared to rotary tillage, SS tillage decreased the plant and ear height by 9.01–9.20 cm and 3.50–4.90 cm, respectively. The stalk dry matter accumulation was enhanced by 8.98%–24.98%, while stalk diameter between two and seven internodes increased by 0.47– 4.15 mm. Stalk cellulose increased by 11.83% –12.38%, hemicellulose increased by 6.7%–15.97%, and lignin increased by 9.86%–15.9%. The rind puncture and crushing strength improved by 3.11%–20.06% and 11.90%–27.07%, respectively. The bending strength increased by 6.25%–27.96% and the lodging rate decreased by 1.20%–6.04%. Yield increased by 7.58%–8.17%. At SS tillage when density increased, the index changes in ZD2 were mostly less than those in XY335. The rind penetration strength, bending strength, crushing strength, stalk diameter, and dry matter accumulation all had a negative correlation with the lodging rate. It suggested that SS tillage was beneficial to lodging resistance and, in combination with stalk lodging-resistant varieties, can effectively alleviate the problem of stalk lodging after increased planting density.

## 1 Introduction

Maize is one of the world’s and China’s major crops in both plant area and yield and plays a pivotal role in national food security. With the development of the Chinese economy and population growth, the demand for grain has shown a steady increase, and maize output will need to rise to fulfill the needs (Ghose, 2014; Tigchelaar et al., 2018). Increasing planting density and the number of effective harvested ears per unit area is one of the key cultivation measures to increase maize yield (Ma et al., 2014). In China, the average planting density of maize rose from 15,000 plants ha^−1^ in the 1950s to 60,000 plants ha^−1^ in 2010, and the average grain yield elevated from 1,000 kg hm^−1^ in the 1940s to 6,000 kg hm^−1^ in 2010 (Li et al., 2016). In recent years, the planting density of maize in China has grown from 99,500 plants ha^−1^ to 105,000 plants ha^−1^ (Kamran et al., 2018; Liu et al., 2021; Zhang et al., 2022).

Increasing planting density is an effective approach to increase maize yield with minimum input. However, as density increases, increased competitive pressure between plants changes the structure and function of maize plants and populations, thus affecting maize stalk morphology, carbohydrate accumulation and distribution, chemical components, root morphology and structure, and mechanical strength of stalks, and increasing the risk of lodging (Xue et al., 2020a; Sher et al., 2018; Zheng et al., 2021; Qi et al., 2023). Stalk lodging causes annual yield loss of up to 75% (Li et al., 2015) and reduces the quality of grain production (Da et al., 2016), becoming an important concern in modern maize production. The direct mechanized harvest and grain drying into storage can simplify the production link, improve production efficiency, aid in achieving large-scale production, and help realize progressive advancement in agricultural practices (Xue et al.,2018a). However, the high lodging rate hinders the large-scale popularization and application of whole-mechanized processes. Grain loss due to straw fall is higher than root fall because straw fall disrupts the transport of photosynthetic products, nutrients, and water (Shah et al., 2021). Lodging at silking and milk-ripening stages (R1 and R3) influence grain filling and yield, while stalk lodging (R6) at the dewatering stage after physiological maturity adversely impacts mechanical yield (Xie et al., 2022). Morphological characteristics of the plant, including plant height and ear height, and mechanical strength of the stalk, including crushing strength (CS) and bending strength (BS), are often strong indicators of maize stalk resistance, and these indicators are often closely related to tillage practices (Shi et al., 2016; Li et al., 2023).

Tillage affects soil structure, moisture, fertility, gases, heat, and other factors that influence crop root growth and yield formation. However, there is little information on how maize stalk traits respond to tillage practices and their effects on maize stalk resistance to lodging. Deep tillage (e.g., subsoiling, deep ploughing, or ripping, to a depth > 20 cm) has been shown to enhance the structure and health of compacted soils (Hamza and Anderson, 2005; Hall et al., 2010). Tillage affects various soil physical, biological, and chemical properties and thus also impacts crop yield. Previous studies have shown that deep tillage improves the soil properties in the tilled layer by reducing soil bulk density and penetration resistance (Varsa et al., 1997), increasing soil porosity, hydraulic conductivity, and infiltration rate (Laddha and Totawat, 1997), and creating a more favorable soil environment for root growth and crop production than shallow tillage. Conventional tillage with soil compaction resulted in barren summer maize, leading to low root stress resistance (Bian et al., 2016). As compared to shallow tillage and no tillage, subsoil (SS) tillage can break up the compacted hardpan layer without inverting it, decrease the soil bulk density and soil compaction, and increase the soil water content. This improves soil structure and, thus, promotes root growth and boosts maize yield (Sun et al., 2018; Xu et al., 2019; Wang et al., 2020b). Root fixation properties and stalk development complement each other, promoting the accumulation of material and mechanical strength formation of aboveground stalks, effectively reducing the rate of lodging. Liu et al. reported that subsoil significantly improved the vascular transport efficiency of the stalk and enhanced stalk strength. On the other hand, it also improves root vigor, facilitates water and nutrient uptake, promotes dry matter accumulation, and increases the mechanical strength of the stalks, ultimately improving resistance to lodging (Liu et al., 2013). Ma et al. demonstrated that subsoil can reduce the ear height of maize under dense planting conditions, slow down the densification pressure, and increase maize stalk puncture strength, CS, and BS to varying degrees, so as to optimize stalk morphology and mechanical traits, which is conducive to improving the resistance to lodging and efficiently accommodating larger populations (Liu et al., 2013; Ma et al., 2014; Zhang et al., 2015). Yu et al. showed that subsoil improves the soil environment, which promotes the growth of the root system and helps to absorb water and nutrients from deeper layers of the soil, and that the use of subsoil technology can further increase the planting density, giving full play to the yield potential of varieties (Yu et al., 2013). It can be seen that SS tillage can be one of the effective ways to exploit the potential of maize for densification and resistance to lodging. Therefore, an in-depth investigation of the effect of SS tillage on maize stalk resistance after densification will be of great significance for the application of SS tillage measures to improve maize resistance in production and for the realization of the full mechanization of maize.

The average plough layer is 18.5 cm in China (Li and Wang, 2010) and lower than 35 cm in the United States (Li and Wang, 2010). At a high density, constructing a reasonable soil topsoil layer can promote material accumulation during the formation of stem mechanical strength, and thus reduce the lodging risk. Therefore, it was hypothesized that SS tillage could improve the lodging resistance of maize stalks under high-density conditions. The present study was carried out to (1) elucidate the responses of plant morphology, dry matter accumulation, and mechanical characteristics to subsoiling tillage; and (2) evaluate the response of different maize varieties to SS tillage at different densities. The study’s findings provide ideas and technical assistance for the mechanized harvesting of maize as well as the densification and yield increase.

## 2 Materials and methods

### 2.1 Experimental site

During 2019–2020, the experiment was conducted in the Modern Agriculture Expo Park of China at Tu met Right Banner, Baotou City, Inner Mongolia (40°28′ 28′′, 110°29′ 5′′). The previous crop was maize and the soil type was sandy loam. **Table 1** reflects the main meteorological elements in the growing period, while soil nutritional contents are listed in **Table 2**.

**Table 1:** Main meteorological factors during the growth period in the experimental area.

Month	Precipitation (mm)		Average temperature (°C)		Solar radiation (W/m^2^)	
	2019	2020	2019	2020	2019	2020
4	14.8	0.8	11.8	9.8	509.8	547.5
5	12.5	20.0	17.8	17.5	563.3	575.7
6	80.8	23.6	23.4	22.8	487.1	588.6
7	57.1	112.0	23.6	22.2	475.0	540.0
8	57.1	129.8	21.3	20.5	465.0	443.3
9	95.0	45.8	3.8	15.5	384.1	401.8
10	4.2	7.2	12.3	5.9	296.5	347.0

**Table 2:** Physical properties of the soil in the test area.

Years	Total nitrogen (g/kg)	Alkaline N (mg/kg)	Alkaline K (mg/kg)	Alkaline P (mg/kg)	Organic matter (g/kg)	pH
2019	1.31	79.31	144.00	18.45	17.74	8.50
2020	1.54	74.89	136.28	21.32	17.83	8.36

### 2.2 Experimental design

The experiment was conducted utilizing a re-split zone experiment design with three factors (**Table 3**): tillage method, planting density, and variety. In the main area of cultivation, two treatments were set: subsoil (SS) (35 cm) and traditional rotary tillage (RT) (15 cm);, the total area of the plot is 2,160 m^2^. The sub-plot included a density of 75,000 plants ha^−1^ (D1) (plot area, 180 m^2^; length, 30 m; width, 6 m; row space, 60 cm; and plant space, 22.22 cm) and 105,000 plants ha^−1^ (D2) (plot area, 180 m^2^; length, 30 m; width, 6 m; row space, 60 cm; and plant space, 15.87 cm). Two varieties with different lodging resistance were selected, namely, Xian yu335 (XY335) and Zhong dan2 (ZD2). Three replicates per treatment were established (15 m × 6 m, 90 m^2^ plots). The plots were subjected to continuous tillage on the same site with the same plots in 2019 and 2020.

**Table 3:** Experimental design.

Main plot	Tillage	
	Subsoil (SS)	Rotary tillage (RT)
	35 cm	15 cm
Sub-plot	Density (plant ha^−1^)	
	75,000 (D1)	105,000 (D2)
	Row space 60 cm, plant space 22.22 cm	Row space 60 cm, plant space 15.87 cm
Sub-sub-plot	Varieties	
	Xianyu335 (XY335)	Zhongdan2hao (ZD2)
	Lodging resistance	Weak lodging resistance

The sowing dates were 23 April 2019– and 26 April 2020–. Nitrogen fertilizer (225.0 kg hm^−2^), P_2_O_5_ (150.0 kg hm^−2^), and K_2_O (150.0 kg hm^−2^) were used in the test. Nitrogen fertilizer was applied at the jointing, V12, and silking stages in a ratio of 3:6:1, respectively. P_2_O_5_ and K_2_O were used as base fertilizers at one time. RT is representative of the typical tillage practice in the study area, and the soil was tilled to a depth of 20 cm with a rotary cultivator in the spring. The SS treatment was sub-soiled to a depth of 35 cm with an interval of 60 cm using a subsoiler in the autumn and tilled to a depth of 20 cm with a rotary cultivator in the spring. The growth period was irrigated four times: pre-sowing, V12, R1, and R3 stage, with 750 m^3^ hm^−2^ irrigation water. Other management was the same as in field production.

### 2.3 Sampling and measurements

#### 2.3.1 Field stalk lodging rate

Field lodging of maize was evaluated at the silking stage (R1), 30 days after spinning (R3), physiological maturity stage (R6), and 15 days after ripening (R6–15d).


Stalk lodging rate (%)=stalk lodging number/total plant number×100%

#### 2.3.2 Plant morphological indexes

Plant height (from the ground to the top of the tassel) and ear height (from the ground to the node bearing the ear) were measured using a ruler. The stalk diameter (d) was measured using a Vernier caliper at the midpoint of the internode at its narrowest side (d1) and widest side (d2). It was calculated as d = (d1+d2)/2.


Ear ratio (%)=Ear height/Plant height×100%

#### 2.3.3 Sampling and determination of internode mechanical strength

At the R1 stage, three maize plants were randomly chosen from each treatment. The stalks were cut from the ground, and leaves and other parts were removed, followed by sorting and marking according to the nodes. The mechanical indexes of the stalks were determined as follows:

Rind Puncture Strength (RPS): The SY-S03 probe (1 mm^−2^) was slowly pressed perpendicular to the stalk at a constant rate to read the maximum penetration (N mm^−2^) of the stalk epidermis. It was measured at the base of the second to seventh internodes.

Crushing strength (CS): The SY-S03 type stalk strength measuring instrument was used. The 1 cm^−2^ probe perpendicular to the stalk axis was used to press slowly and evenly in the middle of the internode with the pressure head of the testing machine so that it could reach the CS value (N cm^−2^) when it was torn. It was measured at the base of the third, fifth, and seventh internodes.

Bending strength (BS): The 0.5 cm^−2^ probe of the SY-S03 stalk strength measuring instrument was perpendicular to the stalk axis, and the pressure head of the testing machine was pressed in the middle of the internode at a slow and uniform speed so that its bending reached the maximum value (N cm^−2^). It was measured at the base of the second, fourth, and sixth internodes.

#### 2.3.4 Stalk dry matter accumulation

A total of four periods were measured, which were at the silking stage (R1), 30 days of silking (R3), physiological maturity stage (R6), and 15 days after maturity (R6–15d); the stalk samples were intercepted and placed in the oven at 105°C for 30 min, dried at 80°C, and weighed dry.

### 2.4 Data and statistical analysis

Data were processed using Microsoft Excel 2019 (Microsoft, Inc., Redmond, WA, USA). The statistical software SAS 9.4 (SAS Institute Inc., Raleigh, CA, USA) was used for data variance and correlation analysis. One-way ANOVA was used to compare the differences between treatments each year at the same growth stage. Three-way ANOVA was used to assess the effects of individual factors and interactions between factors on the indicators. The significance test was performed using LSD (least significant difference) and Duncan’s method. Pearson’s correlation coefficient was used for correlation analysis. Additionally, figures were created using Sigma plot 12.5.

## 3 Results

### 3.1 Lodging rate of the stalks

ANOVA results (**Table 4**) indicated that maize stalk lodging rates varied significantly based on density, variety, tillage, density × variety, and tillage × variety. After 15 days of maturity, the lodging rate of maize under the SS plot was 2.55% and 2.01% points lower than RT in 2019 and 2020, respectively. The planting density increased from D1 to D2, resulting in a significant increase in lodging rate, which climbed by 10.18% and 3.61% points in 2019 and 2020, respectively, at maturity. At 15 days after maturity, it elevated by 11.23% and 3.76% points. XY335 had a lower lodging rate than ZD2. **Table 5** shows that, after increasing the density, the lodging rate of ZD2 increased by 1.67%–21.30% under the RT plot, while it increased by 2.53%–18.67% under the SS plot. However, in comparison to RT, SS with increased density exhibited a reduction in lodging rate ranging from 0.40% to 6.49%. Thus, employing SS may minimize the maize lodging rate despite the increase in planting density.

**Table 4:** ANOVA results for maize stalk lodging rate (%) from 2019 to 2020.

Year	2019				2020			
Growth period	R1	R3	R6	R6-15d	R1	R3	R6	R6-15d
Tillage								
RT	1.86a	6.29a	11.72a	15.41a	0.41a	5.88a	7.23a	8.12a
SS	1.32a	4.48b	9.47b	12.86b	0.25b	4.72b	5.03b	6.11b
Density								
D1	1.07b	4.79b	5.51b	8.52b	0.15b	4.32b	4.34b	5.23b
D2	2.11a	5.97a	15.69a	19.75a	0.51a	6.27a	7.95a	8.99a
Varieties								
ZD2	3.18a	10.77a	21.00a	25.82a	0.66a	10.60a	12.27a	14.23a
XY335	0.00b	0.00b	0.19b	2.45b	0.00b	0.00b	0.00b	0.00b
Source								
Tillage (T)	ns	**	**	**	*	*	**	**
Density (D)	**	*	*	*	**	**	**	**
T × D	ns	ns	ns	ns	ns	ns	ns	**
Varieties (V)	**	**	**	**	**	**	**	**
T × V	ns	**	**	**	*	**	**	**
D × V	**	*	*	*	**	**	**	**
T × D × V	ns	ns	ns	ns	ns	ns	ns	**

Different lowercase letters indicate significant differences (p < 0.05) among various treatments. *Significant at the 0.05 probability level. **Significant at the 0.01 probability level. ns indicates non-significance. R1, silking stage; R3, milk stage; R6, physiological maturity; R6-15d, 15 days after physiological maturity. SS, subsoil; RT, rotary tillage; XY335, Xian yu335; ZD2, Zhong dan 2. D1, 75,000 plants ha^−1^; D2, 105,000 plants ha^−1^.

**Table 5:** Lodging rate (%) of maize at different growth periods.

Year	Varieties	Tillage	Density	Growth period			
				R1	R3	R6	R6-15d
2019	ZD2	RT	D1	2.89b	11.61b	12.41c	17.70c
			D2	4.56a	13.56a	33.72a	38.16a
		SS	D1	1.38c	7.59c	9.61d	14.48d
			D2	3.91ab	10.34b	28.28b	32.95b
	XY335	RT	D1	0.00d	0.00d	0.00e	1.35fg
			D2	0.00d	0.00d	0.78e	4.45e
		SS	D1	0.00d	0.00d	0.00e	0.56g
			D2	0.00d	0.00d	0.00e	3.45ef
2020	ZD2	RT	D1	0.41c	9.53c	9.77c	11.25c
			D2	1.21a	13.99a	19.16a	21.23a
		SS	D1	0.21d	7.77d	7.50d	9.69d
			D2	0.82b	11.12b	12.65b	14.75b
	XY335	RT	D1	0.00e	0.00e	0.00e	0.00e
			D2	0.00e	0.00e	0.00e	0.00e
		SS	D1	0.00e	0.00e	0.00e	0.00e
			D2	0.00e	0.00e	0.00e	0.00e

Different lowercase letters indicate significant differences (p < 0.05) among various treatments. R1, silking stage; R3, milk stage; R6, physiological maturity; R6-15d, 15 days after physiological maturity. SS, subsoil; RT, rotary tillage; XY335, Xian yu335; ZD2, Zhong dan 2. D1, 75,000 plants ha^−1^; D2, 105,000 plants ha^−1^.

### 3.2 Plant height, ear height, and ear ratio of maize

ANOVA results showed significant differences in maize plant and ear height across tillage, density, and variety. The plant height varied significantly among tillage × variety, density × variety, and tillage × variety × density.

Subsoil tillage effectively reduced plant and ear height, but there was no significant effect on the ear ratio. In comparison to RT, the plant and ear heights were reduced by 9.39 cm and 5.32 cm, respectively, under SS. From D1 to D2, the plant and ear height increased by 10.09 cm in 2019 and 4.99 cm in 2020. The plant and ear height of XY335 were lower than ZD2 (**Table 6**).

**Table 6:** ANOVA results for plant height, ear height (cm), and ear ratio from 2019 to 2020.

Year	2019			2020		
Indicators	Plant height	Ear height	Ear ratio	Plant height	Ear height	Ear ratio
Tillage						
RT	297.12a	112.71a	0.38a	310.75a	114.47a	0.36a
SS	288.76b	108.27a	0.36a	300.33b	108.21b	0.35a
Density						
D1	288.67b	108.29b	0.37a	299.75b	108.55b	0.35a
D2	297.28a	112.69a	0.37a	311.33a	114.14a	0.36a
Varieties						
ZD2	304.83a	116.79a	0.38a	309.83a	121.14a	0.38a
XY335	281.12b	104.19b	0.36a	301.25b	101.22b	0.33b
Source						
Tillage (T)	**	*	*	**	**	ns
Density (D)	**	*	ns	**	**	ns
T × D	ns	ns	ns	ns	ns	ns
Varieties (V)	**	**	ns	**	**	**
T × V	*	ns	ns	ns	ns	ns
D × V	**	ns	ns	ns	ns	ns
T × D × V	*	ns	ns	ns	ns	ns

Different lowercase letters indicate significant differences (p < 0.05) among various treatments. *Significant at the 0.05 probability level. **Significant at the 0.01 probability level. ns indicates non-significance. The data in this table are all from the silk spinning period (R1). SS, subsoil; RT, rotary tillage; XY335, Xian yu335; ZD2, Zhong dan 2. D1, 75,000 plants ha^−1^; D2, 105,000 plants ha^−1^.

According to **Table 7**, under the RT plot, when the density increased, the plant and ear height grew by 10.66 cm and 5.13 cm for ZD2, while it was raised by 11.63 cm and 7.20 cm for XY335. SS reduced the rise in plant height and ear height of XY335 and ZD2 due to increased density.

**Table 7:** Plant height, ear height (cm), and ear ratio from 2019 to 2020.

Year	Tillage	Varieties	Density	Plant height	Ear height	Ear ratio
2019	RT	ZD2	D1	303.67 ± 1.25b	113.67 ± 1.25ab	0.38 ± 0.01ab
			D2	311.67 ± 1.9a	118.17 ± 6.46a	0.39 ± 0.02a
		XY335	D1	278.47 ± 0.83f	102.33 ± 1.25de	0.37 ± 0abc
			D2	290.07 ± 0.54d	110.33 ± 3.47bc	0.38 ± 0.01ab
	SS	ZD2	D1	294.83 ± 0.84c	112.33 ± 0.94ab	0.37 ± 0abc
			D2	304.27 ± 1.38b	116.67 ± 1.25a	0.37 ± 0.01abc
		XY335	D1	272.83 ± 0.85g	95.27 ± 1.02f	0.36 ± 0c
			D2	283.13 ± 0.84e	102.4 ± 5.53e	0.37 ± 0.01bc
2020	RT	ZD2	D1	308.33 ± 2.49b	120.33 ± 1.43b	0.39 ± 0a
			D2	321.67 ± 1.7a	126.1 ± 0.83a	0.39 ± 0a
		XY335	D1	300.67 ± 4.19cd	101.07 ± 1.3e	0.34 ± 0.01bc
			D2	312.33 ± 3.09b	107.47 ± 1.11d	0.34 ± 0b
	SS	ZD2	D1	298.67 ± 6.65cd	114.6 ± 1.92c	0.38 ± 0a
			D2	310.67 ± 4.03b	120.6 ± 0.43b	0.39 ± 0.01a
		XY335	D1	291.33 ± 1.25d	98.5 ± 1.63e	0.32 ± 0c
			D2	300.67 ± 0.47cd	105.6 ± 0.43cd	0.34 ± 0.02bc

Different lowercase letters indicate significant differences (p < 0.05) among various treatments. The data in this table are all from the silk spinning period (R1). SS, subsoil; RT, rotary tillage; XY335, Xian yu335; and ZD2, Zhong dan 2. D1, 75,000 plants ha^−1^; D2, 105,000 plants ha^−1^.

### 3.3 Diameter of the second to seventh internodes

ANOVA results (**Table 8**) revealed that the diameter from the second to seventh internodes varied significantly among density, variety, tillage, and density × variety. SS increased diameter from the second to seventh internodes, respectively. When the density increased from D1 to D2, the diameter from the second to seventh internodes decreased significantly. The diameter of the second to seventh internodes of XY335 is larger than that of ZD2. **Table 9** depicts that the stalk diameter between the second and seventh nodes at the base of the maize plant decreases as the node ascends and diminishes with increasing planting density. Under the RT plot, after the increase in density, the reduction in stalk diameter ranged from 9.04% to 17.47% for XY335 and from 10.71% to 22.32% for ZD2. Conversely, under the SS plot, the reduction in stalk diameter was in the range of 2.97%–14.79% for XY335 and 7.43%–17.51% for ZD2. Consequently, it can be inferred that SS mitigates the impact of reduced diameter resulting from increased planting density. Under high density (D2), SS increased diameter in XY335 by 4.65%–20.28% and in ZD2 by 0.72%–16.35% as compared to RT. The higher increase in the diameter of XY335 indicated that it was more receptive to subsoil tillage than ZD2.

**Table 8:** ANOVA results for the diameter (mm) of the second to seventh internodes from 2019 to 2020.

Year	2019						2020					
Internode	Second	Third	Fourth	Fifth	Sixth	Seventh	Second	Third	Fourth	Fifth	Sixth	Seventh
Tillage treatment												
RT	22.09b	20.69b	20.09b	19.36b	18.46b	15.25b	18.25b	18.50b	17.79b	17.69b	16.73b	15.46b
SS	24.64a	22.48a	21.71a	20.92a	19.96a	18.85a	20.21a	21.21a	20.19a	19.50a	18.58a	16.80a
Density												
D1	24.74a	23.04a	22.35a	21.45a	20.53a	18.39a	20.53a	21.04a	20.01a	19.81a	18.72a	17.29a
D2	22.00b	20.13b	19.45b	18.84b	17.90b	15.98b	18.22b	18.67b	17.96b	17.38b	16.59b	14.97b
Varieties												
ZD2	22.21b	20.89b	20.31b	19.27b	19.20a	16.55b	18.36b	18.88b	18.33b	18.00b	16.99b	15.48b
XY335	24.52a	22.28a	21.50a	21.01a	19.23a	17.82a	20.39a	20.83a	19.64a	19.19a	18.32a	16.78a
Source												
Tillage (T)	**	**	**	**	**	**	**	**	**	**	**	**
Density (D)	**	**	**	**	**	**	**	*	**	**	**	**
T × D	*	*	*	*	ns	ns	ns	ns	ns	ns	*	ns
Varieties (V)	**	**	**	**	ns	**	**	*	*	**	**	**
T × V	ns	ns	ns	ns	ns	ns	ns	ns	ns	*	ns	ns
D × V	**	**	**	**	**	ns	ns	ns	ns	ns	**	ns
T × D × V	ns	ns	ns	ns	ns	*	ns	ns	ns	ns	ns	*

Different lowercase letters indicate significant differences (p < 0.05) among various treatments. *Significant at the 0.05 probability level. **Significant at the 0.01 probability level. ns indicates non-significance. SS, subsoil; RT, rotary tillage; XY335, Xian yu335; ZD2, Zhong dan 2. D1, 75,000 plants ha^−1^; D2, 105,000 plants ha^−1^.

**Table 9:** The diameter (mm) from the second to seventh internodes.

Year	Tillage	Varieties	Density	Second	Third	Fourth	Fifth	Sixth	Seventh
2019	RT	ZD2	D1	25.04 ± 0.66bc	22.97 ± 0.73bc	22.99 ± 0.42b	21.51 ± 0.39bc	20.74 ± 0.49bc	19.31 ± 0.24b
			D2	21.47 ± 0.03f	20.52 ± 0.23d	20.69 ± 0.78cd	18.85 ± 0.4e	19.2 ± 0.49e	16.42 ± 0.35d
		XY335	D1	28.35 ± 0.71a	25.17 ± 0.45a	25.31 ± 0.3a	23.94 ± 0.68a	22.48 ± 0.35a	21.2 ± 0.5a
			D2	25.71 ± 0.07b	22.95 ± 0.54bc	23.49 ± 0.39b	21.74 ± 0.37bc	21.81 ± 0.6bc	18.8 ± 0.34b
	SS	ZD2	D1	23.43 ± 0.18d	21.87 ± 0.58c	21.47 ± 0.14c	20.75 ± 0.43cd	18.98 ± 0.41cd	17.05 ± 0.22cd
			D2	20.8 ± 0.19f	19.36 ± 0.28c	19.17 ± 0.57e	18.37 ± 0.19e	16.93 ± 0.34e	14.31 ± 0.58f
		XY335	D1	24.7 ± 0.3c	23.65 ± 0.89d	22.71 ± 0.61b	21.96 ± 0.52b	20.98 ± 0.44b	17.29 ± 0.08c
			D2	22.34 ± 0.39e	19.71 ± 0.41d	20.44 ± 0.25d	19.94 ± 0.62d	19.39 ± 0.26d	15.4 ± 0.31e
2020	RT	ZD2	D1	18.95 ± 0.73cd	20.96 ± 0.34b	20.05 ± 0.77b	19.14 ± 0.08cd	18.19 ± 0.57c	16.86 ± 0.71bc
			D2	16.59 ± 0.09e	16.28 ± 0.23d	16.31 ± 0.78e	16.01 ± 0.89f	15.64 ± 0.36e	14.13 ± 0.7d
		XY335	D1	20.98 ± 0.79ab	21.03 ± 0.44b	19.39 ± 0.2bc	19.96 ± 0.63bc	19.02 ± 0.33b	17.84 ± 0.66ab
			D2	18.05 ± 0.58de	18.65 ± 0.57c	17.64 ± 0.3de	17.35 ± 0.52e	16.18 ± 0.33e	14.73 ± 0.36d
	SS	ZD2	D1	21.55 ± 1.1ab	22.26 ± 1.38ab	20.64 ± 0.85b	20.55 ± 0.48ab	19.21 ± 0.22b	17.89 ± 0.11ab
			D2	17.93 ± 1.32de	18.94 ± 0.69c	18.57 ± 0.27cd	18.01 ± 0.42e	17.04 ± 0.09d	14.76 ± 0.5d
		XY335	D1	22.21 ± 0.36a	22.84 ± 1.49a	22.2 ± 0.89a	21.3 ± 0.6a	20.56 ± 0.41a	18.28 ± 0.92a
			D2	20.33 ± 0.41bc	20.8 ± 0.24b	19.34 ± 0.79bc	18.15 ± 0.19de	17.53 ± 0.47cd	16.26 ± 0.41c

Different lowercase letters indicate significant differences (p < 0.05) among various treatments. SS, subsoil; RT, rotary tillage; XY335, Xian yu335; ZD2, Zhong dan 2. D1, 75,000 plants ha^−1^; D2, 105,000 plants ha^−1^.

### 3.4 Rind puncture strength from the second to seventh internodes

ANOVA results indicated that the RPS from the second to seventh internodes varied significantly among density, variety, tillage, density × variety, tillage × variety, and density × variety. In comparison to RT, SS significantly enhanced the RPS from the second to seventh internodes by 2.68–8.71 N mm^−2^ in 2019 and increased by 4.07–12.32 N mm^−2^ in 2020. When the density increased from D1 to D2, the RPS from the second to seventh internodes dropped considerably. The XY335 cultivar had a higher RPS than the ZD2 cultivar (**Table 10**).

**Table 10:** ANOVA results for rind puncture strength (N mm^−2^) from the second to seventh internodes during 2019 to 2020.

Year	2019						2020					
Internode	Second	Third	Fourth	Fifth	Sixth	Seventh	Second	Third	Fourth	Fifth	Sixth	Seventh
Tillage												
RT	61.14a	55.54b	52.16a	48.97a	47.68a	39.73b	43.57b	45.40a	39.55b	31.35b	27.30b	22.74b
SS	69.85a	62.89a	56.72a	51.65a	47.72a	43.15a	55.89a	50.44a	46.42a	40.42a	34.23a	26.81a
Density												
D1	69.40a	63.34a	58.00a	53.38a	49.95a	44.79a	57.98a	53.27a	48.20a	41.28a	35.70a	28.58a
D2	61.60b	55.09b	50.88b	47.24b	43.45b	38.10b	41.48b	42.56b	37.77b	30.49b	25.83b	20.97b
Varieties												
ZD2	61.76b	55.51b	47.44b	47.15b	43.90b	39.89b	47.72b	46.23a	41.00b	35.34a	29.23b	22.73b
XY335	69.23a	62.91a	61.45a	53.46a	49.50a	43.00a	51.74a	49.60a	44.97a	36.43a	32.30a	26.82a
Source												
Tillage (T)	**	**	ns	ns	ns	**	**	ns	**	**	**	**
Density (D)	**	**	**	**	**	**	**	**	**	**	**	**
T × D	ns	ns	ns	ns	ns	**	ns	ns	ns	ns	ns	*
Varieties (V)	**	**	**	**	**	**	*	ns	*	ns	**	**
T × V	*	ns	**	ns	ns	ns	ns	ns	*	ns	ns	ns
D × V	ns	ns	**	ns	ns	*	ns	ns	ns	ns	*	*
T × D × V	ns	ns	ns	ns	ns	ns	ns	ns	ns	ns	ns	ns

Different lowercase letters indicate significant differences (p < 0.05) among various treatments. *Significant at the 0.05 probability level. **Significant at the 0.01 probability level. ns indicates non-significance. SS, subsoil; RT, rotary tillage; XY335, Xian yu335; ZD2, Zhong dan 2. D1, 75,000 plants ha^−1^; D2, 105,000 plants ha^−1^.

**Figure 1:**
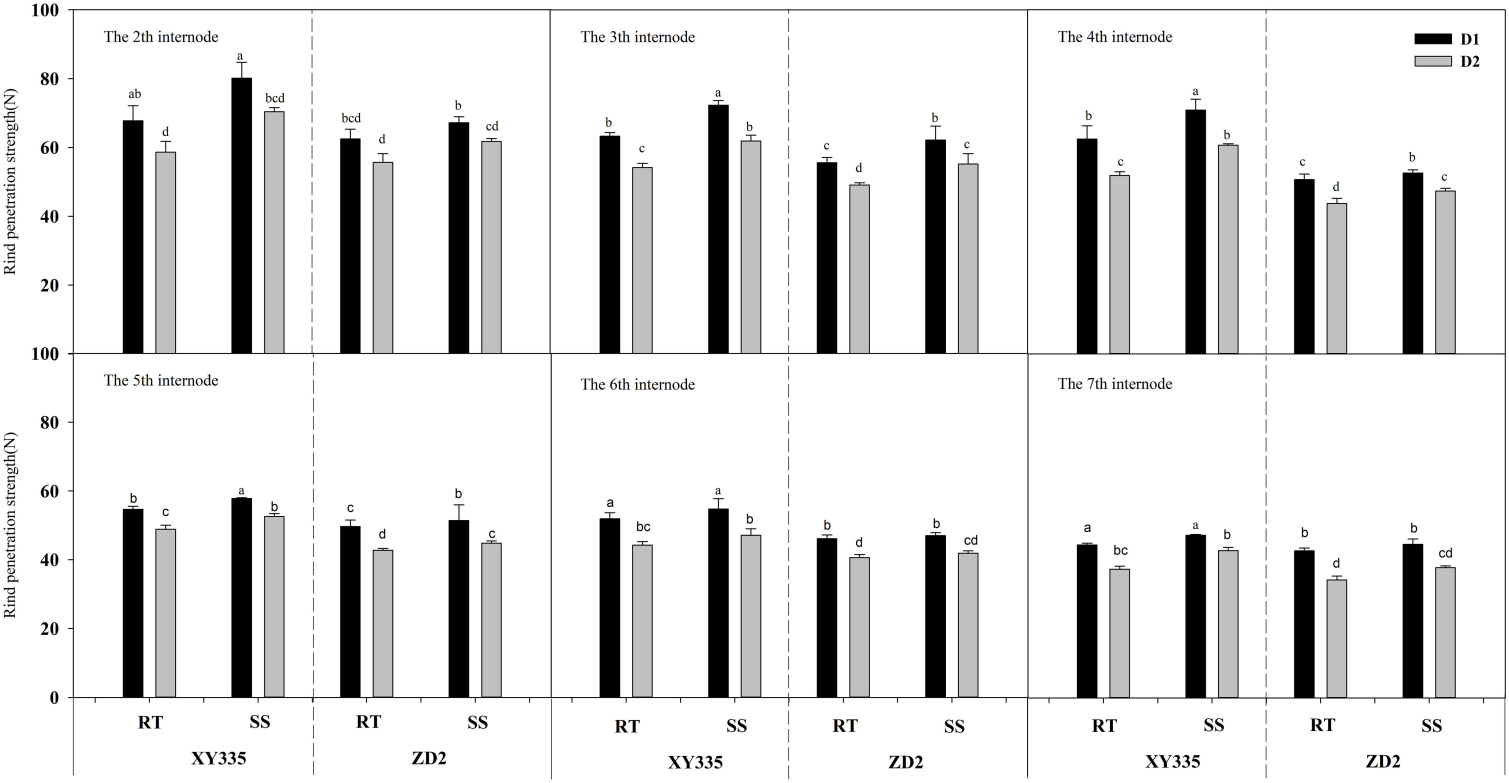
Effect of subsoil tillage and planting density on the penetration strength of maize stalk internodes in 2019. Different lowercase letters indicate significant differences (*p* < 0.05) among various treatments; SS, subsoil; RT, rotary tillage; XY335, Xian yu335; ZD2, Zhong dan 2. D1, 75,000 plants ha^−1^; D2, 105,000 plants ha^−1^.

**Figure 2:**
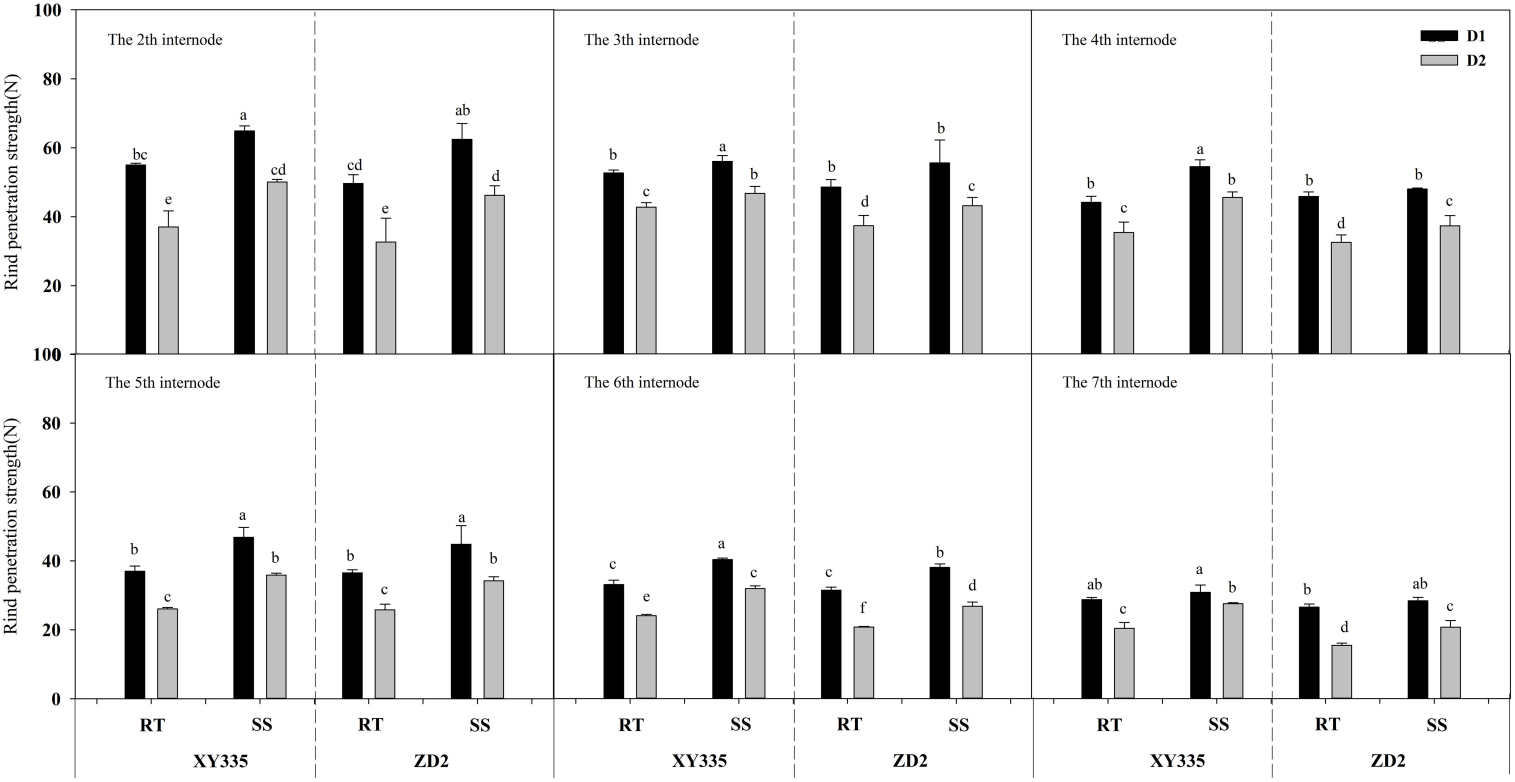
Effect of subsoil tillage and planting density on the penetration strength of maize stalk internodes in 2020. Different lowercase letters indicate significant differences (*p* < 0.05) among various treatments; SS, subsoil; RT, rotary tillage; XY335, Xian yu335; ZD2, Zhong dan 2. D1, 75,000 plants ha^−1^; D2, 105,000 plants ha^−1^.

**Figures 1**, **2** depict that the RPS of the stalk internodes at the base of the maize plant decreases with the upward position of the node and drops significantly as the planting density increases. According to the RT plot, the reduction in RPS after increased density varied from 10.67% to 32.66% for XY335 and from 10.93% to 41.76% for ZD2. In contrast, under the SS plot, the reduction in RPS after increased density was in the range of 9.05%–23.36% for XY335 and 8.18%–29.71% for ZD2, with XY335 exhibiting a more pronounced effect. Consequently, it can be inferred that SS tillage mitigates the decrease in RPS between the second and seventh stalk nodes following higher planting density. Under high density (D2), SS increased XY335 RPS by 6.55%–37.59% and ZD2 by 3.11%–33.91% compared to RT. The larger increase in XY335 indicates that the response of XY335 RPS to subsoil tillage was greater than that of ZD2.

### 3.5 Crushing strength

ANOVA results indicated that the CS of the second, fourth, and sixth internodes differed significantly across density, variety, tillage, tillage × variety, and density × variety. Comparing SS to RT, it significantly increased the CS of the second, fourth, and sixth internodes by 55.7, 55.34, and 37.56 N cm^−2^ in 2019, and by 64.16, 71.2, and 32.67 N cm^−2^ in 2020, respectively. When the density increased from D1 to D2, the CS of the second, fourth, and sixth internodes decreased significantly. The XY335 cultivar had higher CS than the ZD2 cultivar (**Table 11**).

**Table 11:** Variance analysis of the impact of subsoil tillage and planting density on internode crushing strength (N cm^−2^) during 2019–2020.

Year	2019			2020		
Internode	Second	Fourth	Sixth	Second	Fourth	Sixth
Tillage treatment						
RT	432.91b	351.81b	234.17b	361.27b	248.91b	159.39b
SS	488.61a	407.15a	271.73a	425.43a	320.11a	192.06a
Density						
D1	510.81a	433.78a	284.27a	434.95a	310.64a	200.77a
D2	410.72b	325.18b	221.63b	351.75b	258.38b	150.68b
Varieties						
ZD2	443.30b	315.65b	192.50b	377.29b	255.48b	159.2b
XY335	478.23a	443.31a	313.41a	409.41a	313.54a	192.25a
Source						
Tillage (T)	**	**	**	**	**	**
Density (D)	**	**	**	**	**	**
T × D	ns	**	ns	ns	ns	ns
Varieties (V)	**	**	**	**	**	**
T × V	*	*	*	ns	**	*
D × V	**	*	*	ns	*	ns
T × D × V	ns	ns	ns	ns	ns	ns

Different lowercase letters indicate significant differences (p < 0.05) among various treatments. *Significant at the 0.05 probability level. **Significant at the 0.01 probability level. ns indicates non-significance. SS, subsoil; RT, rotary tillage; XY335, Xian yu335; ZD2, Zhong dan 2. D1, 75,000 plants ha^−1^; D2, 105,000 plants ha^−1^.

**Figure 3:**
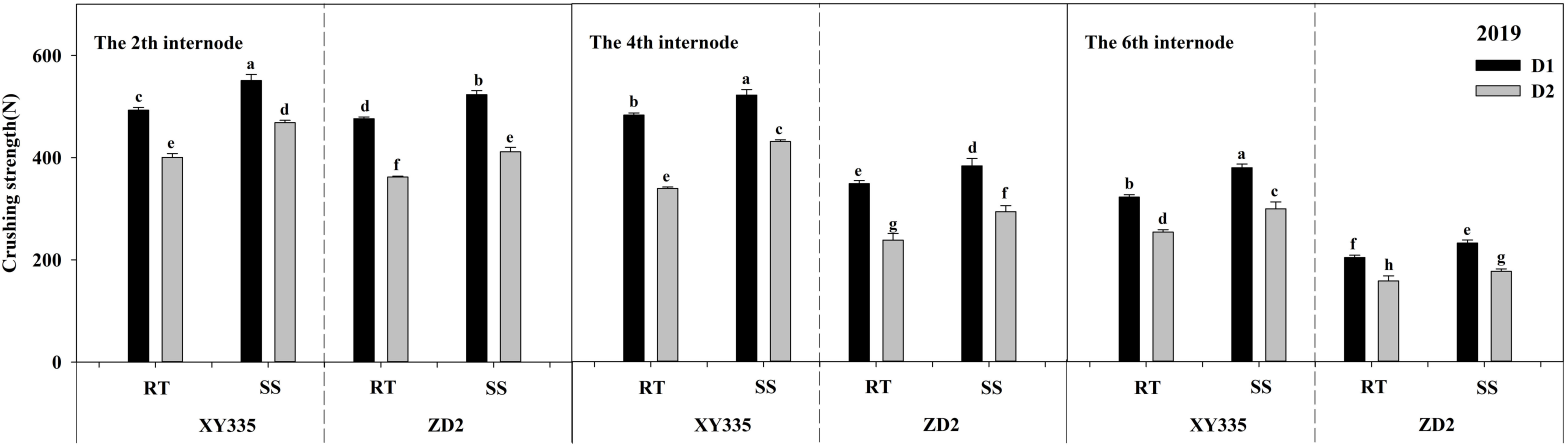
Effect of subsoil tillage and planting density on the crushing strength (N cm^−2^) of maize stalk internodes in 2019. Different lowercase letters indicate significant differences (*p* < 0.05) among various treatments; SS, subsoil; RT, rotary tillage; XY335, Xian yu335; ZD2, Zhong dan 2. D1, 75,000 plants ha^−1^; D2, 105,000 plants ha^−1^.

**Figure 4:**
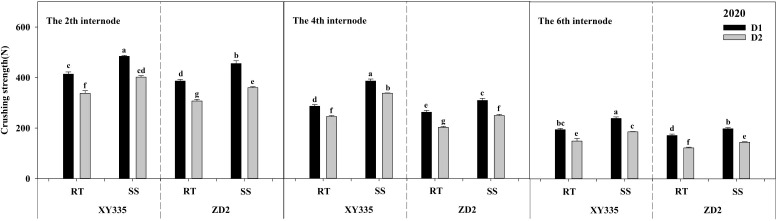
Effect of subsoil tillage and planting density on the crushing strength (N cm^−2^) of maize stalk internodes in 2020. Different lowercase letters indicate significant differences (*p* < 0.05) among various treatments; SS, subsoil; RT, rotary tillage; XY335, Xian yu335; ZD2, Zhong dan 2. D1, 75,000 plants ha^−1^; D2, 105,000 plants ha^−1^.

The CS of maize stalk internodes decreased with the upward position of the node and reduced significantly with increasing planting density, as seen in **Figures 3**, **4**. Under the RT plot, the reduction in CS after increased density ranged from 10.67% to 32.66% for XY335 and from 10.93% to 41.76% for ZD2. Conversely, under the SS plot, the reduction in CS after increased density varied from 9.05% to 23.36% for XY335 and from 8.18% to 29.71% for ZD2, with XY335 exhibiting a more pronounced effect. Consequently, it can be inferred that SS tillage attenuates the decrease in CS between the second, fourth, and sixth stalk nodes following increased planting density. Under high density (D2), SS increased CS by 16.96–37.57% for XY335 and 11.90–23.41% for ZD2 compared to RT. The larger increase for XY335 suggested that changes in its CS responded more favorably to subsoil tillage as compared to ZD2.

### 3.6 Bending strength

The ANOVA results exhibited significant differences in the BS of the third, fifth, and seventh internodes based on density, variety, tillage, tillage × variety, and density × variety. When compared with RT, SS tillage significantly raised the BS of the third, fifth, and seventh internodes by 35.77, 69.72, and 61.52 N cm^−2^ in 2019, and by 59.44, 38.40, and 34.37 N cm^−2^ in 2020. As the planting density progressed from D1 to D2, the BS of the third, fifth, and seventh internodes decreased significantly. The BS of the XY335 cultivar was higher than that of the ZD2 cultivar (**Table 12**).

**Table 12:** Variance analysis of effects of subsoil tillage and planting density on internode bending strength (N cm^−2^) during 2019–2020.

Year	2019			2020		
Internode	Third	Fifth	Seventh	Third	Fifth	Seventh
Tillage treatment						
RT	494.81b	403.28b	291.00b	382.87b	332.03b	185.16b
SS	530.58a	473.00a	352.52a	442.31a	370.43a	219.53a
Density						
D1	542.27a	488.43a	356.86a	469.57a	381.57a	228.89a
D2	483.11b	387.85b	286.66b	355.61b	320.88b	175.81b
Varieties						
ZD2	489.15b	404.39b	295.55b	390.91b	326.95b	189.07b
XY335	536.24a	471.89a	347.97a	434.26a	375.51a	215.62a
Source						
Tillage (T)	**	**	**	**	**	**
Density (D)	**	**	**	**	**	**
T × D	**	ns	ns	ns	ns	ns
Varieties (V)	**	**	**	**	**	**
T × V	*	*	ns	**	*	*
D × V	ns	*	ns	ns	ns	*
T × D × V	ns	ns	ns	ns	ns	ns

Different lowercase letters indicate significant differences (p < 0.05) among various treatments. *Significant at the 0.05 probability level. **Significant at the 0.01 probability level. ns indicates non-significance. SS, subsoil; RT, rotary tillage; XY335, Xian yu335; ZD2, Zhong dan 2. D1, 75,000 plants ha^−1^; D2, 105,000 plants ha^−1^.

**Figure 5:**
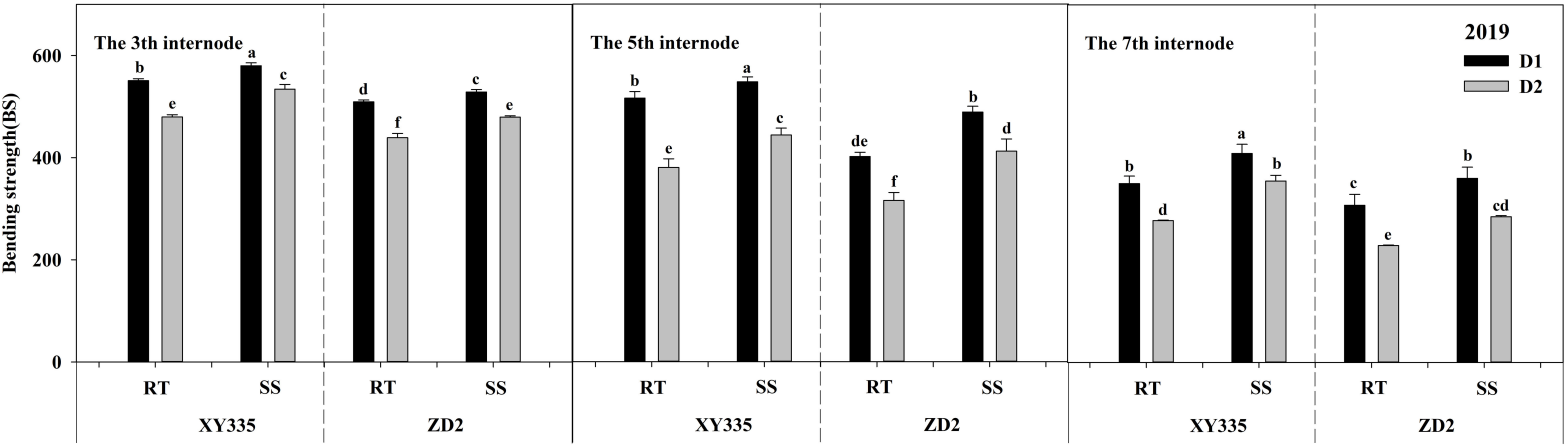
Effect of subsoil tillage and planting density on the bending strength (N cm^−2^) of maize stalk internodes in 2019. Different lowercase letters indicate significant differences (*p* < 0.05) among various treatments; SS, subsoil; RT, rotary tillage; XY335, Xian yu335; ZD2, Zhong dan 2. D1, 75,000 plants ha^−1^; D2, 105,000 plants ha^−1^.

**Figure 6:**
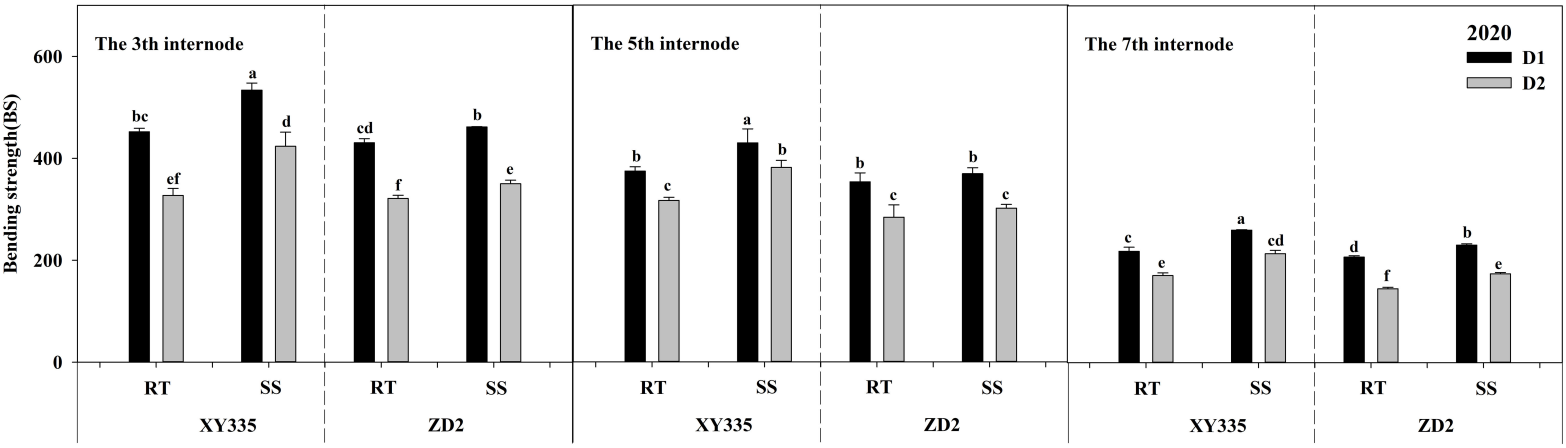
Effect of subsoil tillage and planting density on the bending strength (N cm^−2^) of maize stalk internodes in 2020. Different lowercase letters indicate significant differences (*p* < 0.05) among various treatments; SS, subsoil; RT, rotary tillage; XY335, Xian yu335; ZD2, Zhong dan 2. D1, 75,000 plants ha^−1^; D2, 105,000 plants ha^−1^.

The BS of stalk internodes decreased with the upward position of the node and reduced substantially with increased planting density, as reflected in **Figures 5**, **6**. Under the RT plot, the drop in BS after increased planting density ranged from 12.92% to 27.59% for XY335 and from 13.81% to 30.37% for ZD2. On the other hand, under the SS plot, XY335 showed a more noticeable effect, with the reduction in BS varying from 7.91% to 20.66%, while it ranged from 9.29% to 24.48% for ZD2. Thus, it may be concluded that SS tillage offsets the decrease in BS of the third, fifth, and seventh internodes following increased planting density. Under the high density (D2), SS increased CS by 11.33–27.96% for XY335 and 6.25–26.39% for ZD2 compared to RT. The higher increase for XY335 implied that the BS response of XY335 was more receptive to subsoil tillage than ZD2.

### 3.7 Effects of tillage methods and planting density on stalk dry matter accumulation

The ANOVA results exhibited significant variations in stalk dry matter accumulation (SDMA) per plant depending on density, variety, tillage, tillage × variety, and density × variety. Subsoil tillage substantially raised the SDMA by 4.24–7.37 g in 2019 and by 7.88–10.42 g in 2020, compared with RT. As the planting density progressed from D1 to D2, the SDMA decreased significantly. The XY335 cultivar had greater SDMA than the ZD2 cultivar (**Table 13**).

**Table 13:** Variance analysis of effects of subsoil tillage and planting density on stalk dry matter accumulation (g) in 2019–2020.

Year	2019				2020			
Growth period	R1	R3	R6	R6-15d	R1	R3	R6	R6-15d
Tillage treatment								
RT	54.25b	60.37b	53.18b	45.30b	44.17b	62.22b	53.72b	40.06b
SS	60.63a	66.78a	57.42a	52.67a	54.59a	70.10a	62.83a	48.24a
Density								
D1	64.02a	68.89a	63.32a	55.13a	56.32a	76.41a	71.36a	50.03a
D2	50.86b	58.27b	46.16b	42.84b	42.44b	55.91b	45.19b	38.26b
Varieties								
ZD2	55.12b	57.98b	50.85b	46.61b	46.21b	63.19b	54.36b	41.67b
XY335	59.76a	69.18a	59.74a	51.36a	52.51a	69.13a	62.19a	46.62a
Source								
Tillage (T)	**	**	**	**	**	**	**	**
Density (D)	**	**	**	**	**	**	**	*
T × D	ns	ns	ns	ns	ns	ns	**	ns
Varieties (V)	*	**	**	**	**	**	**	**
T × V	ns	ns	ns	*	ns	ns	**	ns
D × V	*	**	*	ns	ns	ns	**	ns
T × D × V	ns	ns	**	ns	*	ns	**	ns

Different lowercase letters indicate significant differences (p < 0.05) among various treatments. *Significant at the 0.05 probability level. **Significant at the 0.01 probability level. ns indicates non-significance. SS, subsoil; RT, rotary tillage; XY335, Xian yu335; ZD2, Zhong dan 2. D1, 75,000 plants ha^−1^; D2, 105,000 plants ha^−1^.

**Figure 7:**
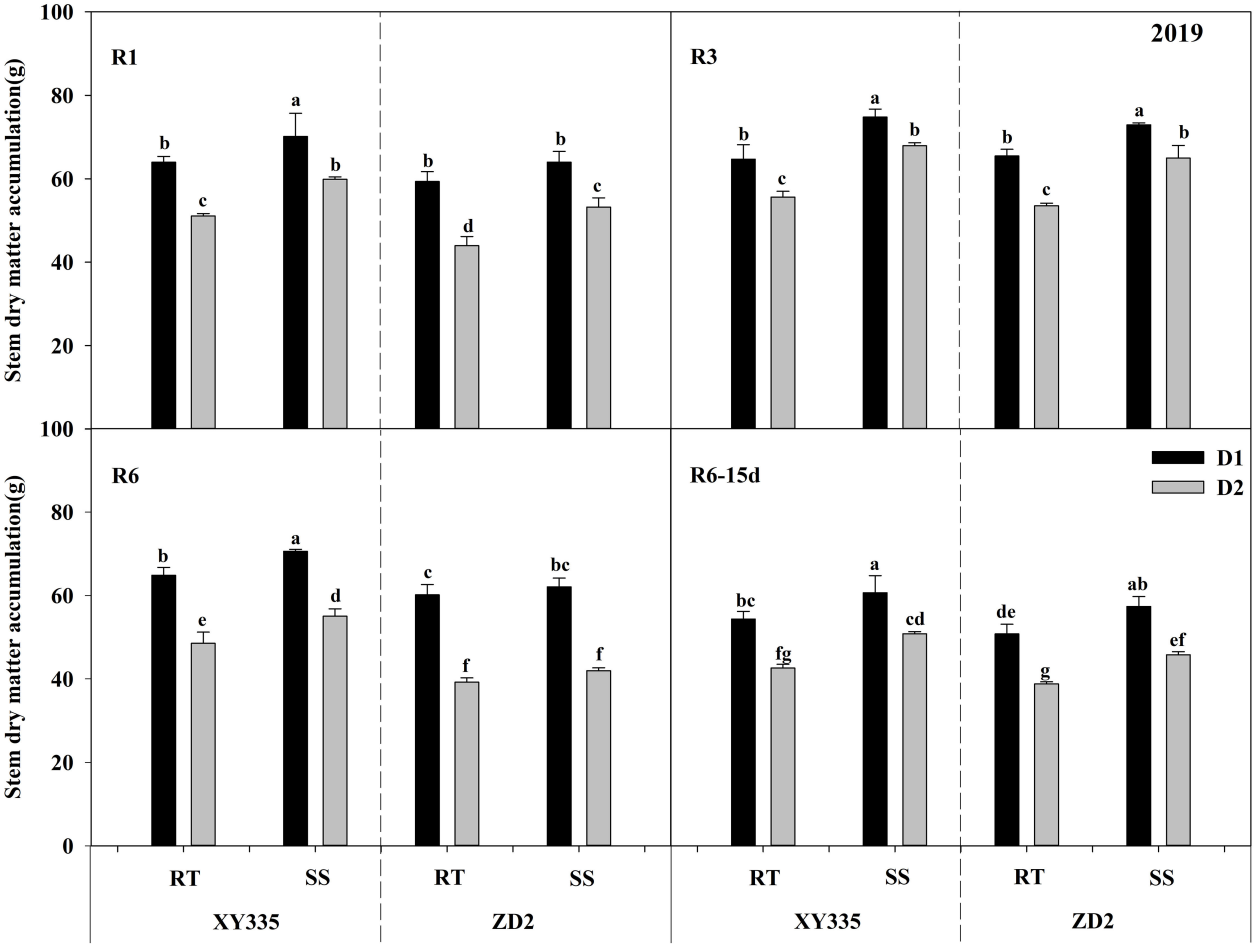
Effect of subsoil tillage and planting density on stalk dry matter accumulation (g) in 2019. Different lowercase letters indicate significant differences (*p* < 0.05) among various treatments; SS, subsoil; RT, rotary tillage; XY335, Xian yu335; ZD2, Zhong dan 2. D1, 75,000 plants ha^−1^; D2, 105,000 plants ha^−1^. R3, milk stage; R6, physiological maturity; R6–15d, 15 days after physiological maturity.

**Figure 8:**
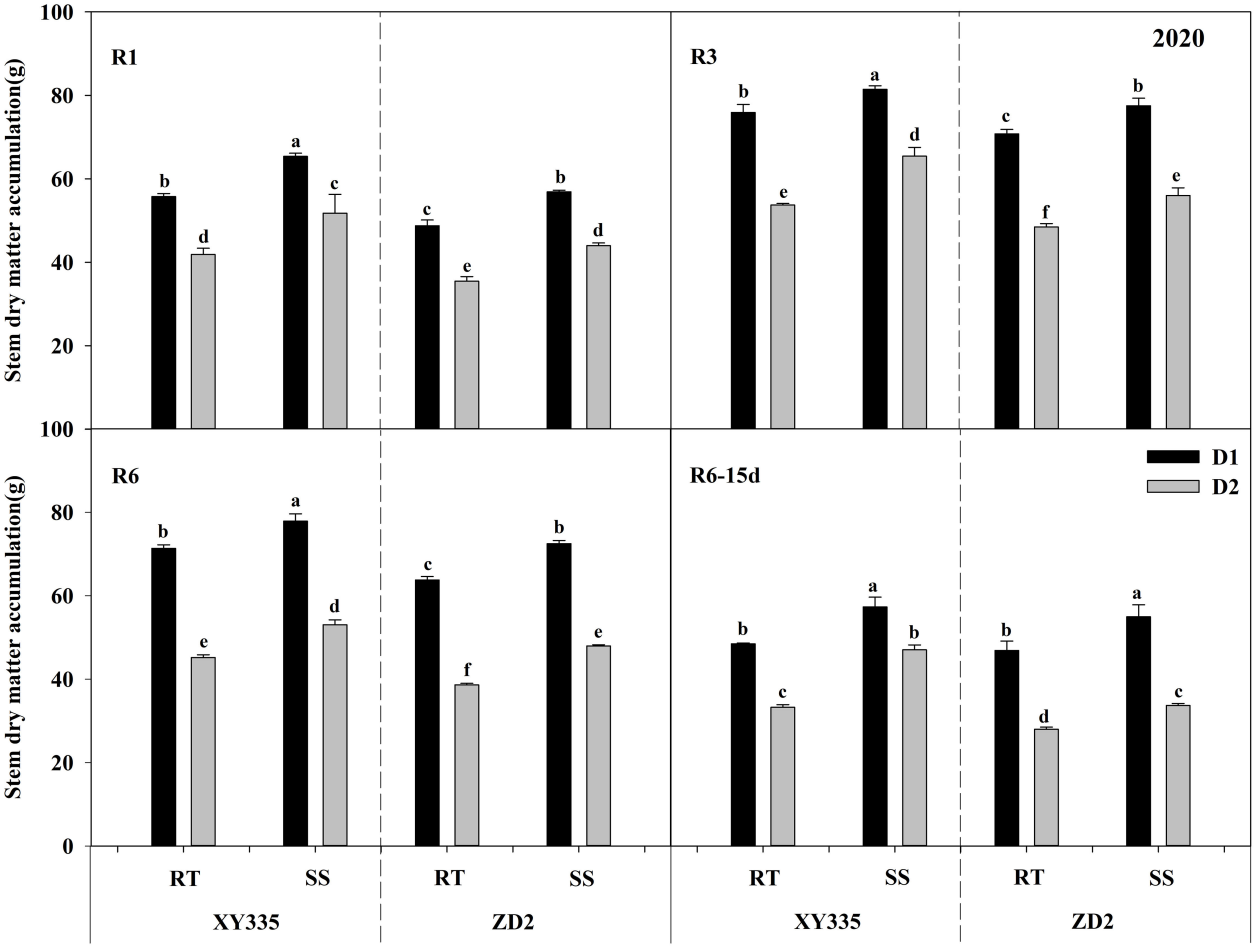
Effect of subsoil tillage and planting density on stalk dry matter accumulation (g) in 2020. Different lowercase letters indicate significant differences (*p* < 0.05) among various treatments; SS, subsoil; RT, rotary tillage; XY335, Xian yu335; ZD2, Zhong dan 2. D1, 75,000 plants ha^−1^; D2, 105,000 plants ha^−1^. R3, milk stage; R6, physiological maturity; R6–15d, 15 days after physiological maturity.

**Figures 7**, **8** depicts that SDMA decreased with increasing planting density. Under the RT plot, SDMA reduced by 17.64%–30.23% for XY335 and 28.28%–41.49% for ZD2 with increasing density. Conversely, under the SS plot, the increase in density decreased SDMA by 11.83%–27.17% for XY335 and 21.70%–33.14% for ZD2. The decrease in SDMA after increased density under SS was lower than that under RT, implying that SS tillage alleviates the decline in SDMA due to the rise in planting density. Under high density (D2), in comparison to RT, SS increased SDMA in XY335 by 13.47%–41.39% and by 7.1%–24.17% in ZD2. The larger increase in SDMA of XY335 suggested that it responded more positively to SS tillage than ZD2.

### 3.8 Correlation between plant morphology, internode mechanical strength, stalk dry matter accumulation, and maize lodging rate

**Figure 9:**
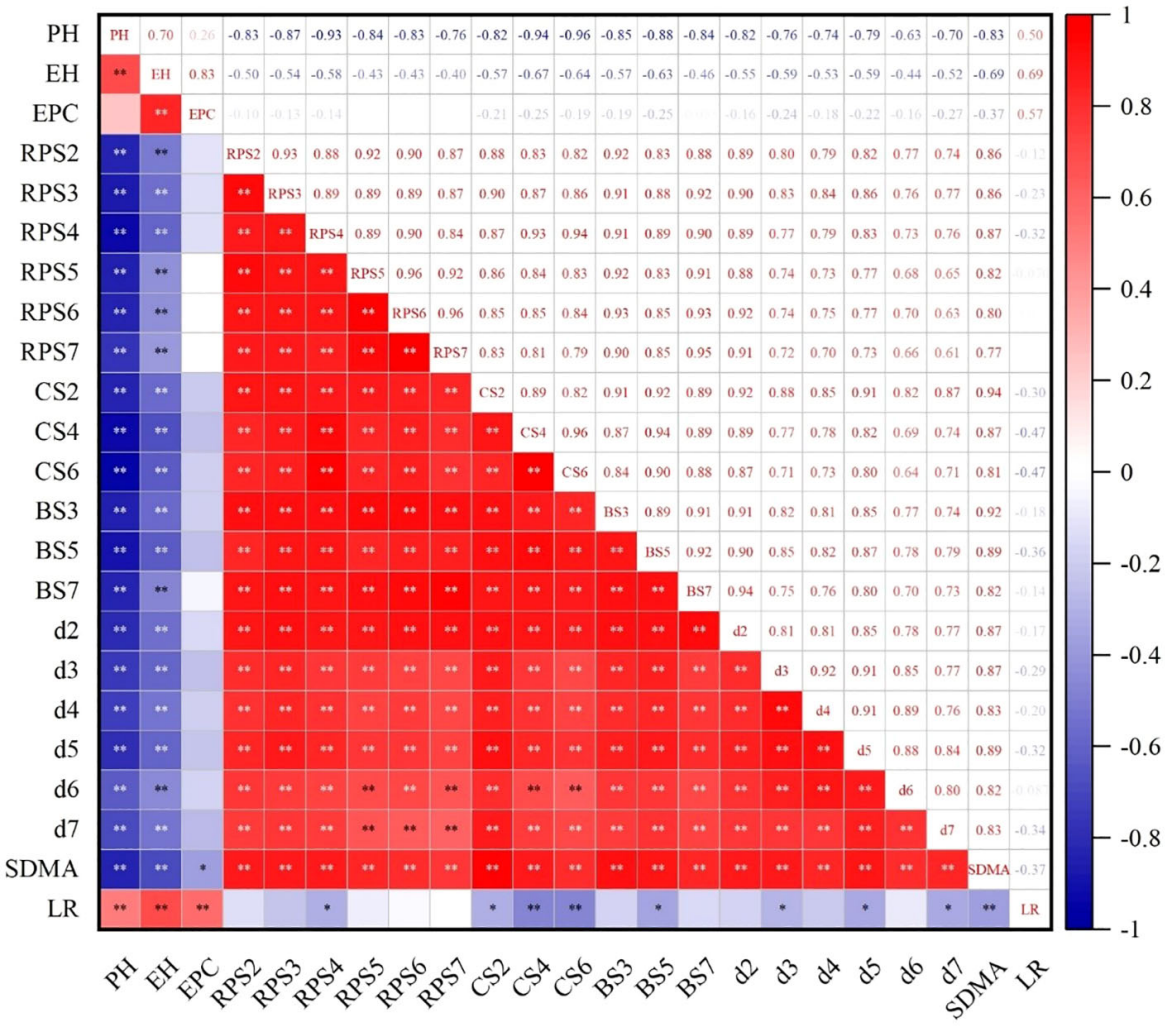
Correlation analysis among indicators. PH, plant height; EH, ear height; ER, ear ratio; BS, bending strength; CS, crushing strength; RPS, rind penetration strength; d, internode diameter; SDMA, stalk dry matter accumulation; LR, lodging rate. ** and *, significances at the 0.01 and 0.05 probability levels, respectively.

As shown in **Figure 9**, plant height, ear height, and ear ratio were significantly and positively correlated with lodging rate. Nevertheless, the RPS of the fourth internode; the CS of the second, fourth, and sixth internodes; the BS of the fifth internode; and the diameter of the third, fifth, and seventh internodes were negatively associated with lodging rate. Furthermore, the CS of the fourth and sixth internodes and accumulation of dry matter of the stalks were negatively and significantly correlated with lodging rate. Significant positive correlations were also found among internode RPS, CS, and BS. There was a significant negative correlation between internode RPS, CS, BS, plant height, and ear height. Additionally, a significant positive correlation was exhibited between SDMA and internode diameter.

### 3.9 Effects of tillage and planting density on stalk internal chemical component content

**Figure 10:**
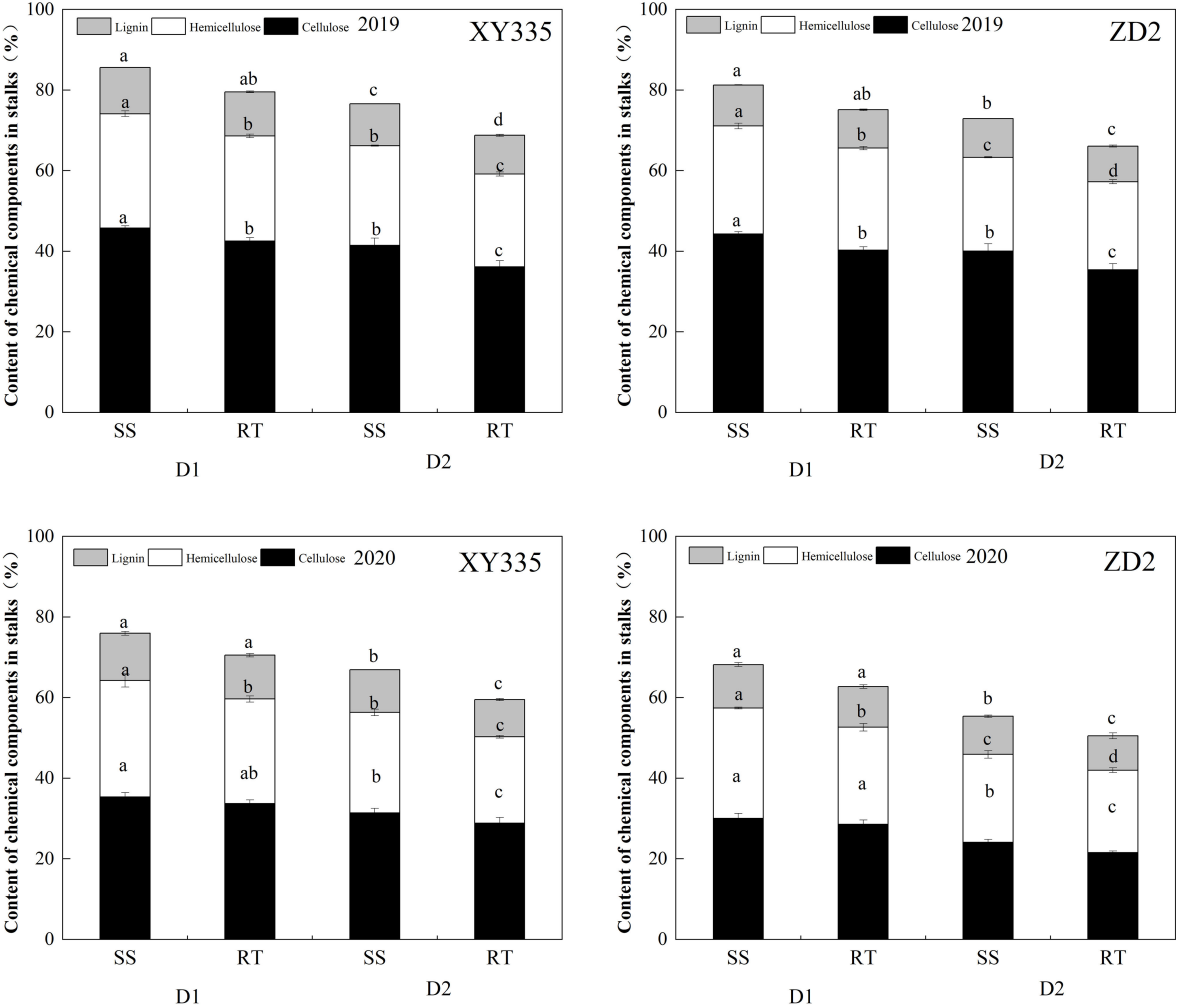
Stalk internal chemical component content under different treatments in 2019–2020. Different lowercase letters indicate significant differences(p < 0.05) among various treatments; SS, subsoil; RT, rotary tillage; XY335, Xian yu335; ZD2, Zhong dan 2. D1, 75,000 plants ha^−1^; D2, 105,000plants ha^−1^.

As can be seen in **Figure 10**, stalk cellulose, hemicellulose, and lignin content decreased significantly at increasing density. Compared to RT, stalk cellulose, hemicellulose, and lignin content increased under SS conditions, which was more significant at D2 density. XY335 and ZD2 cellulose increased by 11.83% and 12.38%, respectively, hemicellulose increased by 15.97% and 6.7%, and lignin increased by 15.9% and 9.86%.

### 3.10 Correlation between stalk chemical content and stalk strength

**Figure 11:**
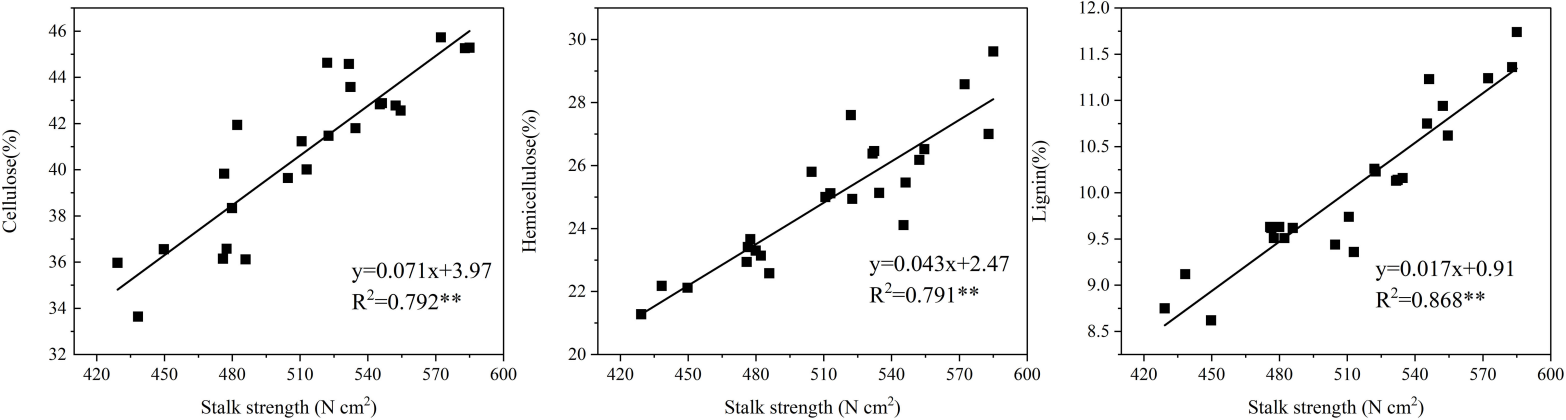
Correlation between stalk chemical content and stalk strength. **indicates significant correlation.

Correlation analysis between the chemical component content in the stalks of maize and stalk bending strength showed that cellulose, hemicellulose, and lignin content were all highly significantly and positively correlated with stalk bending strength (**Figure 11**), with lignin content having the greatest correlation of 0.868 with stalk strength.

### 3.11 Effects of subsoil tillage and planting density on maize yield

**Figure 12:**
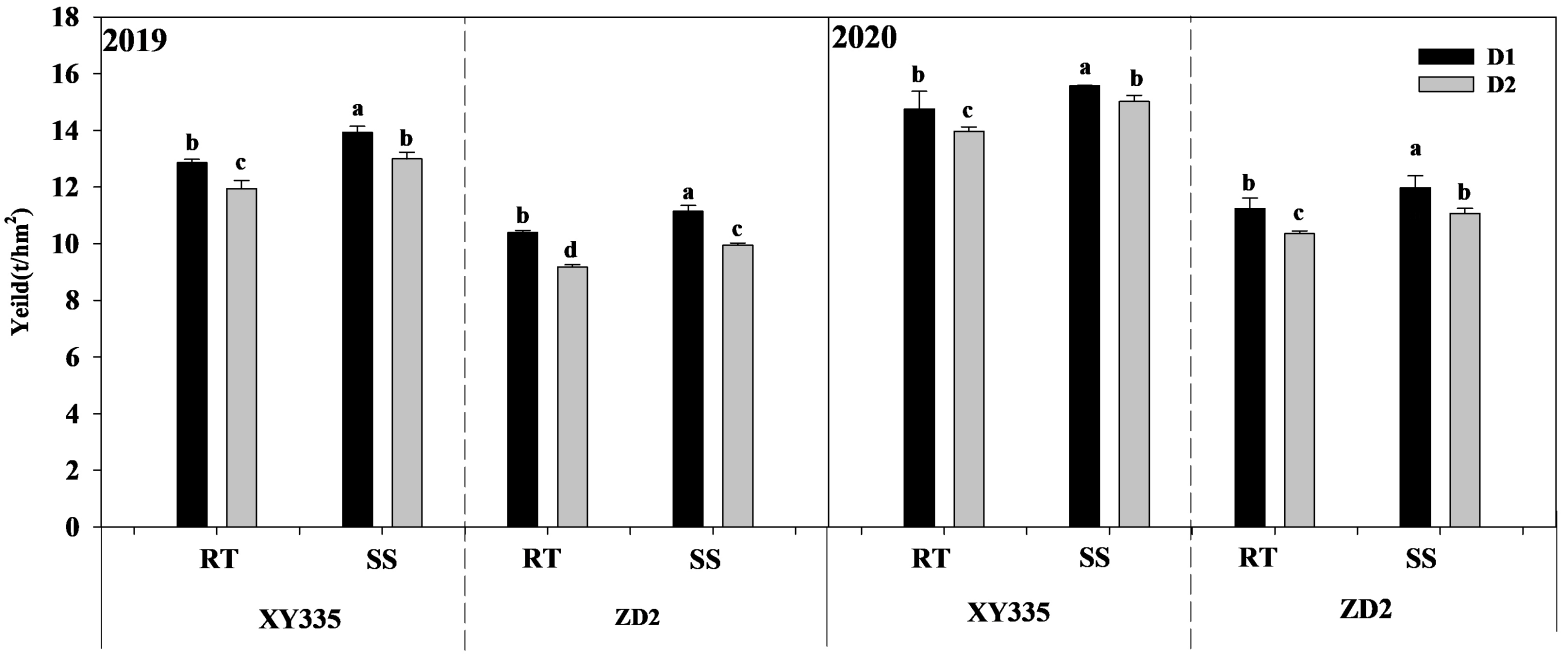
Effect of subsoil tillage and planting density on maize yield (t ha^−1^). Different lowercase letters indicate significant differences(p < 0.05) among various treatments; SS, subsoil; RT, rotary tillage; XY335, Xian yu335; ZD2, Zhong dan 2. D1, 75,000 plants ha^−1^; D2, 105,000plants ha^−1^.

Because the 2-year trend was consistent, the 2-year average was used to describe the results. Under D1 density, compared with RT, the yields of SS condition XY335 and ZD2 increased by 6.92% and 6.84%, and under D2 density, they increased by 8.17% and 7.58%, respectively. The yield increase of SS was greater under high density (**Figure 12**).

## 4 Discussion

Maize crop lodging is detrimental to both yield and quality and is influenced by maize varieties and cultivation practices (Ma et al., 2014). Increased planting density will increase the risk of lodging. Implementing subsoil tillage measures has a substantial impact on reducing lodging. In this study, as the planting density grew, so did maize lodging rates. However, after SS tillage, the lodging rate of ZD2 decreased by 6.04%, indicating alleviation of the lodging pressure induced by increased plant density.

Research on maize stalk lodging has mainly focused on plant morphology, stalk mechanical properties, chemical composition, and anatomical structure, among other aspects (Zhang et al., 2021a). Plant height and ear height (EH) are considered the most crucial morphological features related to stalk lodging. Previous studies have also reported that lowering plant height can enhance a crop’s resistance to lodging (Shekoofa et al., 2008; Peng et al., 2014). Additionally, maize with high resistance to stalk lodging should also have a lower ear position to decrease its center of gravity (Shah et al., 2021). Xie et al. (2022) concluded that reducing the ear ratio significantly decreased the length of internodes below the ear, thereby improving the lodging resistance of maize stalks in China over the past 70 years. Furthermore, maize lodging is closely associated with SDMA (Xue et al., 2020b), stalk diameter, and mechanical properties of the stalk (Xue et al., 2020a). Currently, enhancing stalk mechanical strength, stalk diameter, RPS, and the unit length basal internode dry weight (DWUL) are considered important approaches to improve maize lodging resistance (Robertson et al., 2014; Xue et al., 2018b; Wang et al., 2020a). The significant positive correlation between plant lodging resistance to lignin content and its related enzyme activities and mechanical properties has been generally confirmed (Zhang et al., 2014; Sekhon et al., 2020). In this study, the correlation analysis indicated a significant negative correlation between plant height, ear height, and stalk lodging rate. Stalk thickness, SDMA, RPS, BS, and compressive strength exhibited a significant positive correlation with lodging rate. There was a significant positive correlation between lignin, hemicellulose, and cellulose within the stalks and the mechanical strength of the stalks. These findings are in basic agreement with previous studies.

In this study, as the planting density grew, the plant and ear height of maize increased. Compared to RT, SS tillage could reduce both plant and ear height, thereby lowering the center of gravity of maize. A lower center of gravity is more favorable for the lodging resistance of maize stalks (Shah et al., 2021). With increasing density, the stalk internode diameter, SDMA, and mechanical strength of stalk internodes decreased. In comparison to RT, SS practices could increase the stalk internode diameter and SDMA, enhancing internal lignin and cellulose content of the stalks, thus increasing the mechanical strength of the stalks. This effect was more pronounced under high-density conditions, and the response of lodging-resistant varieties to SS tillage was more evident. Moreover, under SS conditions, the negative impact of high-density stress on stalk dry matter and mechanical strength was less than that under shallow rotation. This indicates that SS tillage can attenuate the negative effects of high-density stress on lodging-related indicators of stalk resistance.

SS tillage can improve the lodging problem caused by high-density planting, possibly because subsoil practices effectively break the plough pan, reduce soil volume (Yu et al., 2023), and decrease soil bulk density and compaction (Lou et al., 2021). This enhances soil porosity and cultivation thickness (Wang et al., 2019), altering soil physical properties and establishing a reasonable tillage layer structure. This is more conducive to the downward growth of maize roots, leading to variations in maize root characteristics. Compared to shallow rotation, subsoiling techniques are more favorable for the growth of maize roots, thereby increasing root biomass and anchorage capacity (Sun et al., 2017). Additionally, SS tillage can enhance root vitality, facilitate water and nutrient absorption, and delay root aging (Yin et al., 2021). A strong root system could increase root anchorage and absorptive capacity for water and nutrients, leading to high yield and resistance to root lodging (Manzur et al., 2014). Root development and stalk development complement each other, and well-developed roots promote the accumulation of stalk material and the formation of mechanical strength, ultimately improving maize lodging resistance.

Previous studies have shown that SS tillage is advantageous for regulating soil nutrients and breaking up soil compaction (Zhang et al., 2021b). Bian et al. (2016) reported that under shallow rotation, soil compaction caused poor root development in summer maize, leading to low lodging resistance of summer maize. Moreover, SS tillage can increase maize emergence rate, plant height, and uniformity, and enhance leaf area index (LAI), affecting photosynthetic characteristics. SS tillage enhances the photosynthetic rate, stomatal conductance, and transpiration rate, and promotes the accumulation of aboveground biomass (Wu et al., 2020). Various studies have supported that SS tillage significantly improves the transport efficiency of stalk vascular bundles, influences maize stalk characteristics, strengthens stalk strength, and ultimately enhances lodging resistance (Liu et al., 2013). SS tillage can also increase soil water storage (Brunel-Saldias et al., 2018; Zhou et al., 2019). As maize reaches the high-growth period, the demand for water increases and soil water storage may decrease. SS tillage is more conducive to the downward movement of water after irrigation or rainfall, effectively increasing moisture content in the deep soil layer (Wang et al., 2019), promoting the formation and accumulation of plant biomass. These factors could contribute to improving maize lodging resistance.

## 5 Conclusion

Subsoil tillage improved the maize lodging resistance, and its effects were more pronounced under high planting density. The lodging rate of maize was significantly negatively correlated with plant height, ear height, and ear position coefficient. It also significantly negatively correlated with stalk diameter, mechanical strength of the base internode, and biomass accumulation. The CS at the fourth and sixth internodes exhibited relatively high correlation coefficients with the lodging rate. Subsoil increases the mechanical strength of stalks by increasing the internal cellulose, hemicellulose, and lignin content of the stalks. SS tillage under high density reduces plant and ear height while elevating stalk diameter, dry matter accumulation, and the mechanical strength of the stalk, leading to an effective reduction in the maize lodging rate. In combination with stalk lodging-resistant varieties (XY335), SS tillage can effectively alleviate the problem of stalk lodging after intensive planting. The findings of this study provide new ideas for improving maize’s resistance to lodging.

## Data availability statement

The raw data supporting the conclusions of this article will be made available by the authors, without undue reservation.

## Author contributions

XF: Data curation, Formal analysis, Investigation, Writing – original draft, Writing – review & editing. DM: Conceptualization, Methodology, Writing – review & editing. TL: Investigation, Writing – review & editing. SH: Investigation, Writing – review & editing. XY: Project administration, Writing – review & editing. JG: Project administration, Writing – review & editing.
